# Video-Assisted Thoracoscopic Sympathectomy for Palmar Hyperhidrosis: A Meta-Analysis of Randomized Controlled Trials

**DOI:** 10.1371/journal.pone.0155184

**Published:** 2016-05-17

**Authors:** Wenxiong Zhang, Dongliang Yu, Han Jiang, Jianjun Xu, Yiping Wei

**Affiliations:** Department of Cardiothoracic Surgery, The Second Affiliated Hospital of Nanchang University, Nanchang, China; Baylor College of Medicine, UNITED STATES

## Abstract

**Objectives:**

Video-assisted thoracoscopic sympathectomy (VTS) is effective in treating palmar hyperhidrosis (PH). However, it is no consensus over which segment should undergo VTS to maximize efficacy and minimize the complications of compensatory hyperhidrosis (CH). This study was designed to compare the efficiency and side effects of VTS of different segments in the treatment of PH.

**Methods:**

A comprehensive search of PubMed, Ovid MEDLINE, EMBASE, Web of Science, ScienceDirect, the Cochrane Library, Scopus and Google Scholar was performed to identify studies comparing VTS of different segments for treatment of PH. The data was analyzed by Revman 5.3 software and SPSS 18.0.

**Results:**

A total of eight randomized controlled trials (RCTs) involving 1200 patients were included. Meta-analysis showed that single segment/low segments VTS could reduce the risk of moderate/severe CH compared with multiple segments/high segments. The risk of total CH had a similar trend. In the subgroup analysis of single segment VTS, no significant differences were found between T2/T3 VTS and other segments in postoperative CH and degree of CH. T4 VTS showed better efficacy in limiting CH compared with other segments.

**Conclusions:**

T4 appears to be the best segment for the surgical treatment of PH. Our findings require further validation in more high-quality, large-scale randomized controlled trials.

## Introduction

Primary palmar hyperhidrosis (PH) is a condition marked by excessive perspiration beyond physiologic need, and is aggravated during periods of stress and anxiety [1, 2]. Video-assisted thoracic sympathectomy (VTS) is currently used worldwide in the treatment of PH. This technique has become popular because of the low morbidity and mortality, and the benefits of minimally invasive surgery offered to the patients [3, 4]. Compensatory hyperhidrosis (CH) is the most common side effect of VTS and has a negative impact on patient’s postoperative quality of life [5]. The relationship of CH to the degree of sympathectomy has been the subject of intense debate among thoracic surgeons [6], partly due to the lack of large-scale clinical research in the area. To determine which segment of the nerve gives the best results post VTS, we performed a meta-analysis of randomized clinical trial data in this area.

## Materials and Methods

### Search strategy

The study was conducted according to the Preferred Reporting Items for Systematic Reviews and Meta-Analyses criteria (PRISMA) as shown in S1 File. A MEDLINE search was performed by two investigators, independently and in duplicate, to identify all relevant scientific literature published up to January 2016. The search included the following databases: PubMed, Ovid MEDLINE, Embase, Web of Science, ScienceDirect, The Cochrane Library, Scopus and Google Scholar. The MeSH terms “hyperhidrosis”, “sympathectomy or sympathicotomy” and “comparative study” were used.

The following types of trial were included: (1) randomized controlled trials (RCTs); (2) trials compared VTS in different segments when treating patients with palmar hyperhidrosis. When data or data subsets were reported in more than one article, the article with the most detail or the most recent article was chosen. Case-only designs, case reports, systematic reviews, meta-analyzes, animal studies, or studies with duplicated data were excluded.

### Data extraction

Two investigators independently extracted data from all eligible studies. The data included first author, year of publication, study design, surgical segments, number of patients per group, follow-up period, evaluation time of CH, clinical outcomes (postoperative resolution of symptoms), satisfied cases and postoperative degree of CH.

### Quality assessment of included studies

Two investigators independently assessed the quality of each study using the Jadad scale [7]. This scale is scored according to the presence of three key methodological features: randomization, blinding and accountability of all patients, including withdrawals and dropouts. Studies are considered good quality if they score ≥ 3 points.

### Statistical analysis

The data were analyzed using Review Manager 5.3 software (The Nordic Cochrane Centre, The Cochrane Collaboration, Copenhagen, Denmark), SPSS 18.0 (International Business Machines Corporation, Armonk, USA) and STATA 12.0 (StataCorp. LP, College Station, Texas, USA). For continuous outcomes, we calculated the mean difference weighted by the inverse of the variance. For dichotomous variables, the relative risk was calculated between two groups with 95% confidence intervals. The Cochrane Q and *I*^2^ statistics were included to evaluate heterogeneity. A fixed-effects model was adopted if heterogeneity was acceptable (*p* > 0.10, or *p* ≤ 0.10 but *I*^2^ ≤ 50%); otherwise, a random-effects model was adopted. A two-tailed p of ≤ 0.05 was deemed statistically significant.

## Results

### Search results and quality assessment of included studies

**Fig 1:**
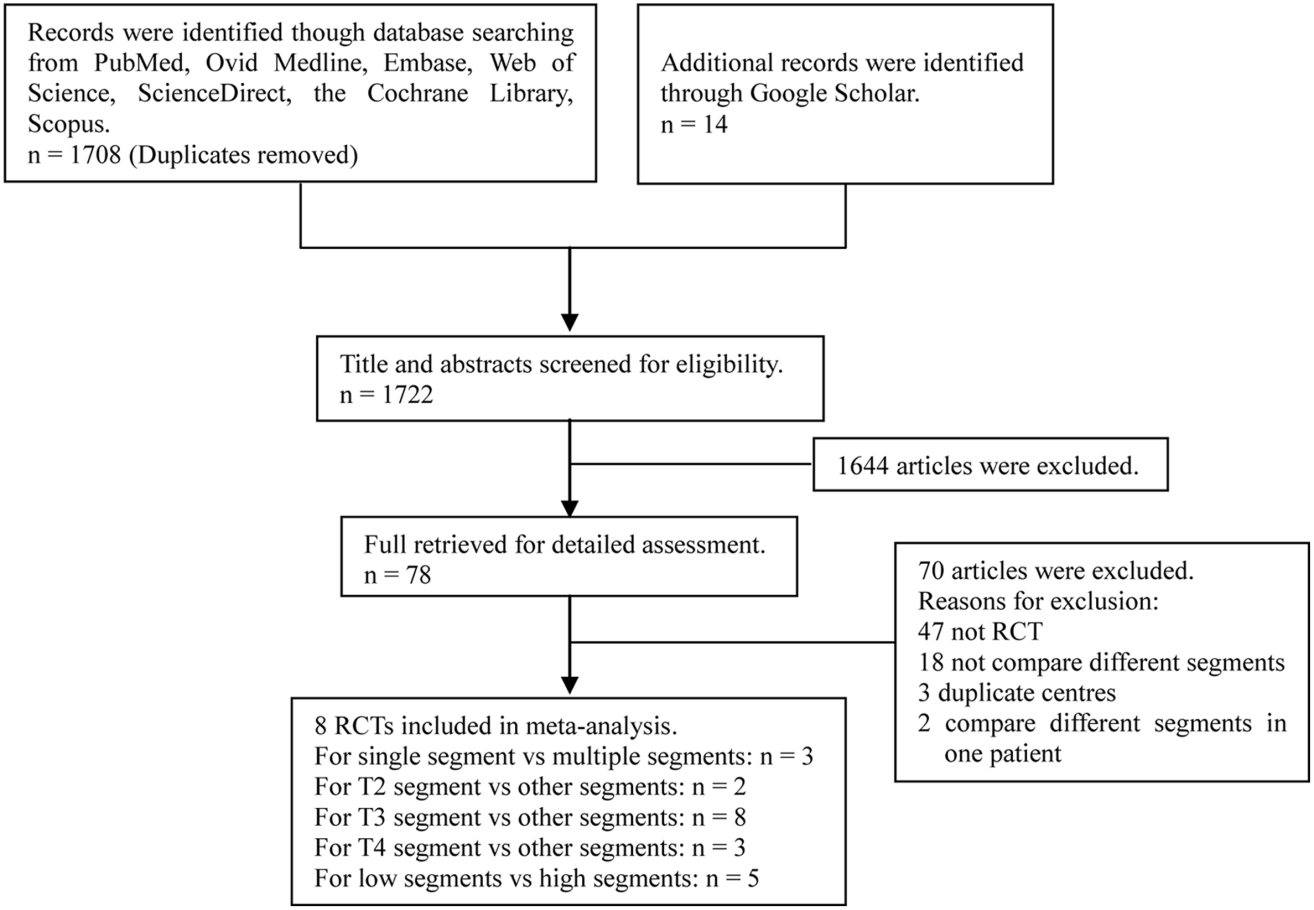
Flow diagram of screened and included papers.

Of the 1722 publications initially identified from the database and reference list searches, we selected eight RCTs for final analysis (Fig 1). The articles involved a total of 1200 patients, with a male:female ratio of 597:603. All patients underwent a bilateral sympathectomy in one stage. According to the Jadad scale, all eight articles were all of good quality, and the baseline characteristics of these articles are listed in Table 1.

**Table 1:** Summary of the eight studies included in the present meta-analysis.

Study		Segments	NO. of Patients (n)	Mean follow-up (mo)	Cases with resolution of symptoms (n)	Evaluation time of CH (mo)	Postoperative CH (n)	Postoperative moderate/severe CH (n)	Satisfied cases (n)	Quality (score)
2005	Yazbek [8]	T2	30	6	30	6	30	10	N/A	4
		T3	30		29		29	3	N/A	
2006	Lin M [9]	T2-T4	207	21.2	207	N/A	58	22	96.10%	3
		T3	131		131		7	2	100.00%	
		T3-T4	60		60		4	2	100.00%	
2007	Yang [10]	T3	78	13.8	78	1	55	18	N/A	3
		T4	85		85		38	6	N/A	
2008	Li [11]	T2-T4	115	12	115	3	33	18	89.60%	4
		T3	117		117		25	13	96.60%	
2009	Liu [12]	T3	68	18.5	62	6	48	9	100.00%	3
		T4	73	17.2	69		39	2	100.00%	
2011	Ishy [13]	T3	20	12	20	12	20	1	N/A	4
		T4	20		20		15	1	N/A	
2011	Baumgartner [14]	T2	61	12	58	12	40	4	100.00%	4
		T3	60		57		29	3	97.50%	
2012	Fiorelli [15]	T2-T3	23	6	21	6	1	0	100.00%	4
		T3	22		21		0	0	100.00%	

We evaluated three aspects of the operation: (1) resolution of symptoms; (2) patient satisfaction; (3) postoperative complications. In the meta-analysis, we found no significant difference in “resolution of symptoms” and “patient satisfaction” between different segments (*p* > 0.05). The data suggest that VTS of all segments could improve the symptom of hyperhidrosis, with a satisfaction close to 100% in all segments. CH was the most common complication reported and, when severe, could significantly affect quality of life. In the present study, we took postoperative degree of CH as the main index to evaluate the success of the operation.

### Comparison of single segment and multiple segments

**Fig 2:**
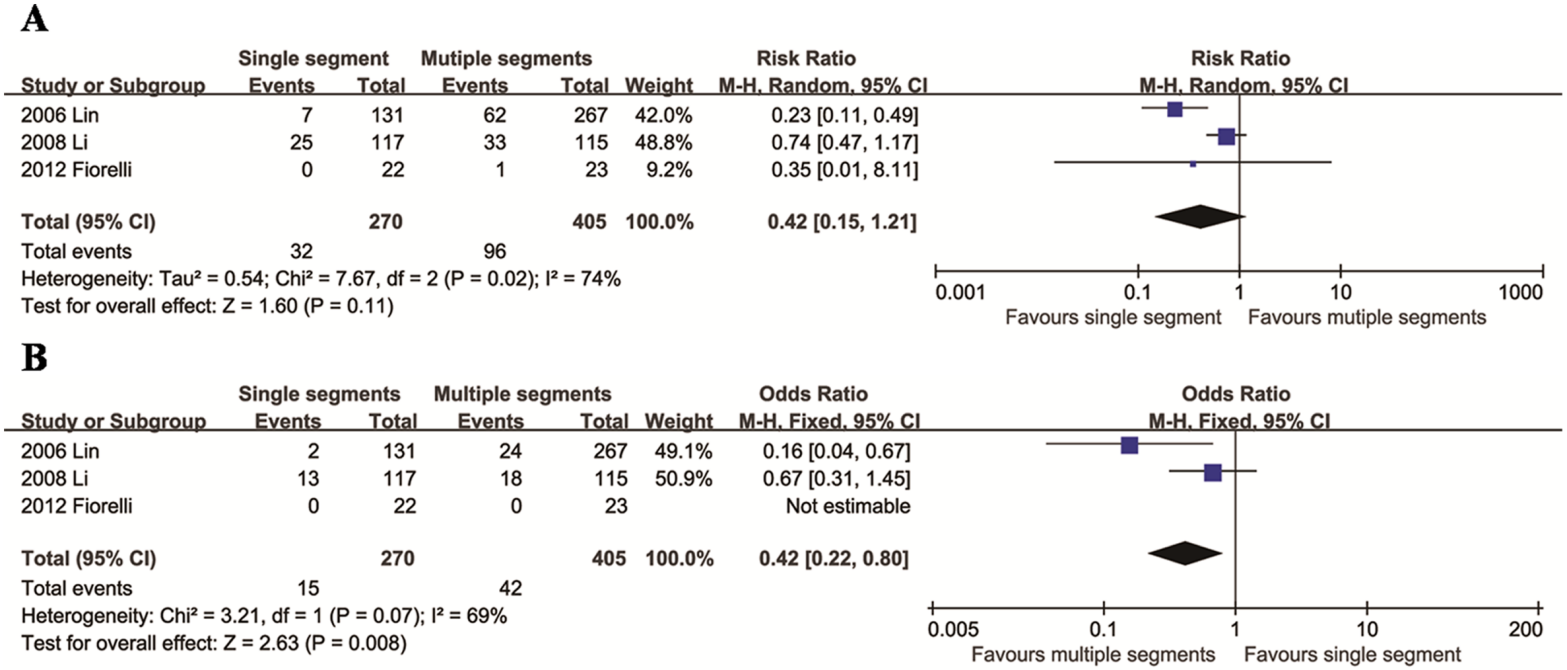
Relative risks for total CH (A) and moderate/severe CH (B) in the single segment group compared to the multiple segment group.

We identified three articles to compare VTS of a single segment and multiple segments. The mean difference in total CH between the two groups was not significant (95% confidence interval [CI]: 0.15 to 1.21, *p* = 0.11), with significant heterogeneity across studies (*p* = 0.02; *I*^2^ = 74%, Fig 2A). More severe CH was found in the multiple segments group (95% CI: 0.22 to 0.80, *p* = 0.008), with significant heterogeneity across studies (*p* = 0.07; *I*^2^ = 69%, Fig 2A).

### Comparison of T2 segment with other segments

**Fig 3:**
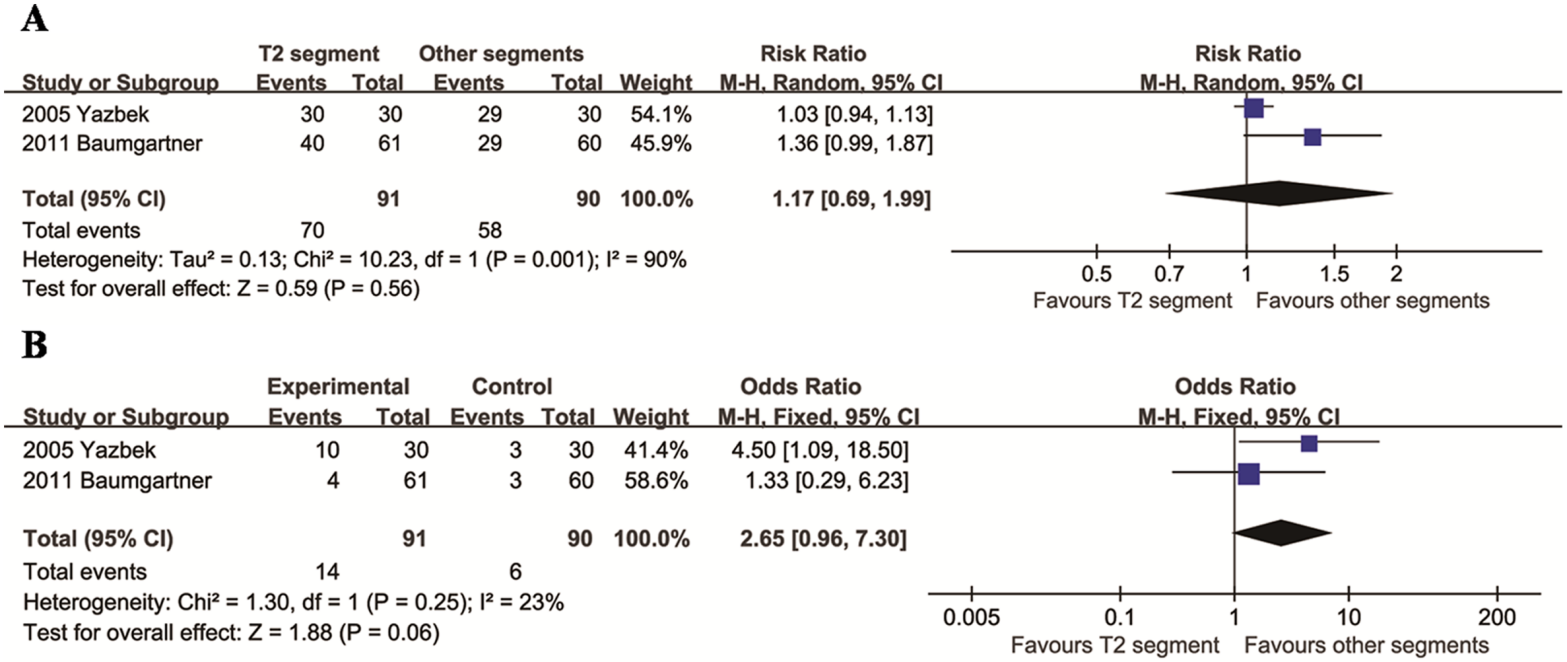
Relative risks for total CH (A) and moderate/severe CH (B) in the T2 segment group compared to the other segments group.

We identified two articles to compare TVS of the T2 segment and other segments. The mean difference in total CH between the two groups was not significant (95% CI: 0.69 to 1.99, *p* = 0.56), with significant heterogeneity across studies (*p* = 0.001; *I*^2^ = 90%, Fig 3A). The mean difference in degree of CH between the two groups was not significant (95% CI: 0.96 to 7.30, *p* = 0.06), with acceptable heterogeneity across studies (*p* = 0.07; *I*^2^ = 69%, Fig 3B).

### Comparison of T3 segment with other segments

**Fig 4:**
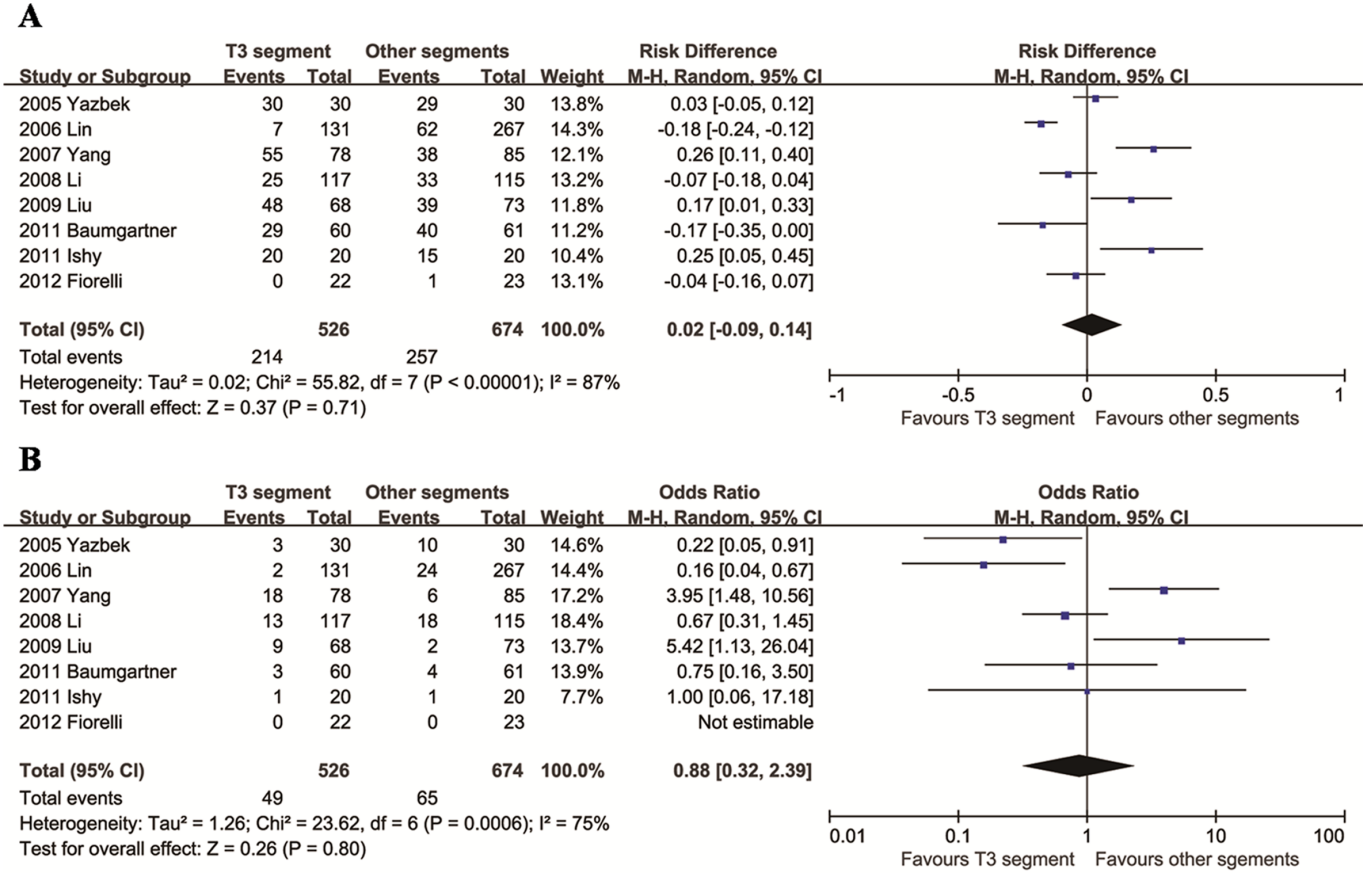
Relative risks for total CH (A) and moderate/severe CH (B) in the T3 segment group compared to the other segments group.

We identified eight articles that compared T3 segment with other segments. The mean differences between the two groups in both comparisons (total CH and degree of CH) were not significant (*p* > 0.05), with significant heterogeneity across studies (*p* < 0.001; *I*^2^ > 70%, Fig 4).

### Comparison of T4 segment with other segments

**Fig 5:**
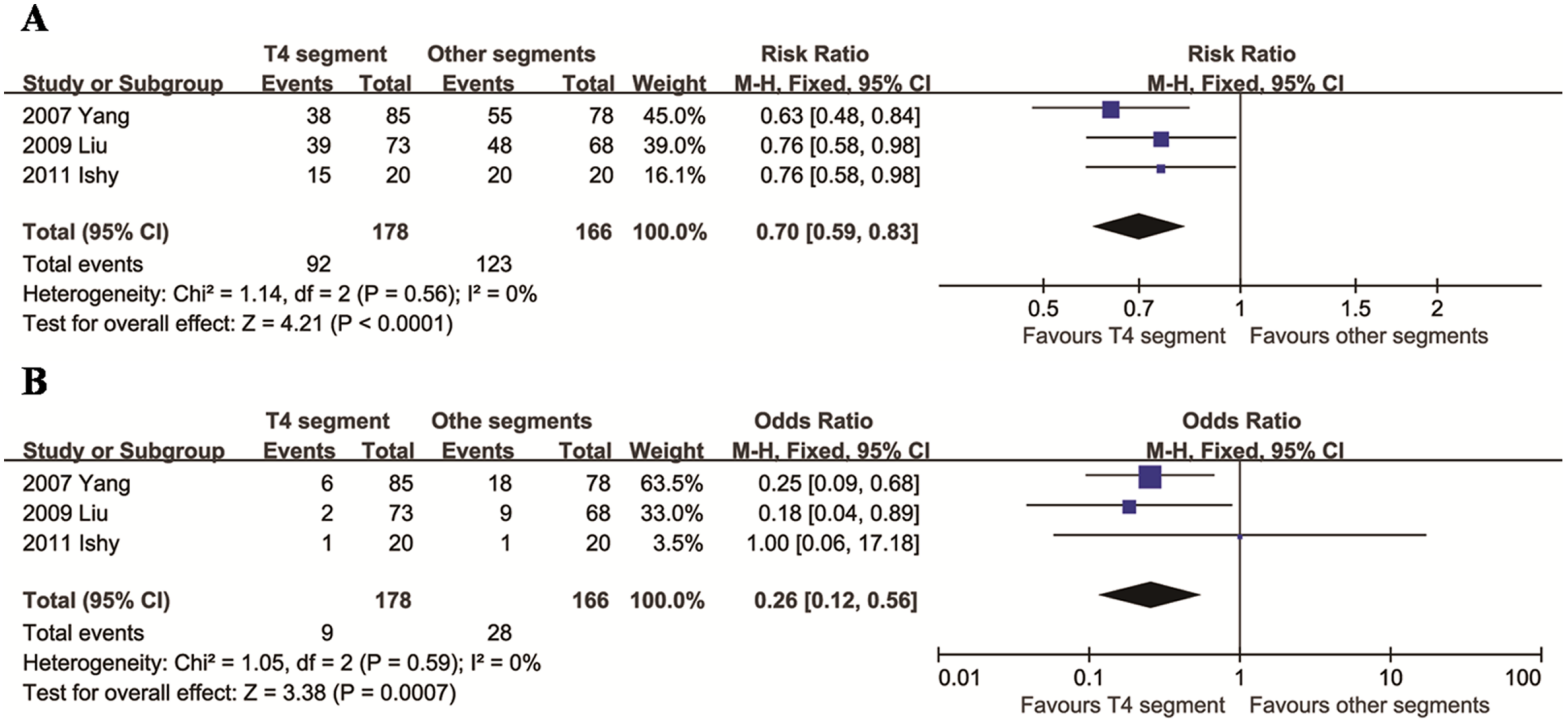
Relative risks for total CH (A) and moderate/severe CH (B) in the T4 segment group compared to the other segments group.

We identified three articles that compared VTS of the T4 segment and other segments, all of which compared T4 and T3. There was no evidence of heterogeneity between these studies (*I*^2^ = 0%). The results showed fewer total CH and moderate/severe CH in the T4 segment group (*p* < 0.001, Fig 5).

### Comparison of low and high segments

**Fig 6:**
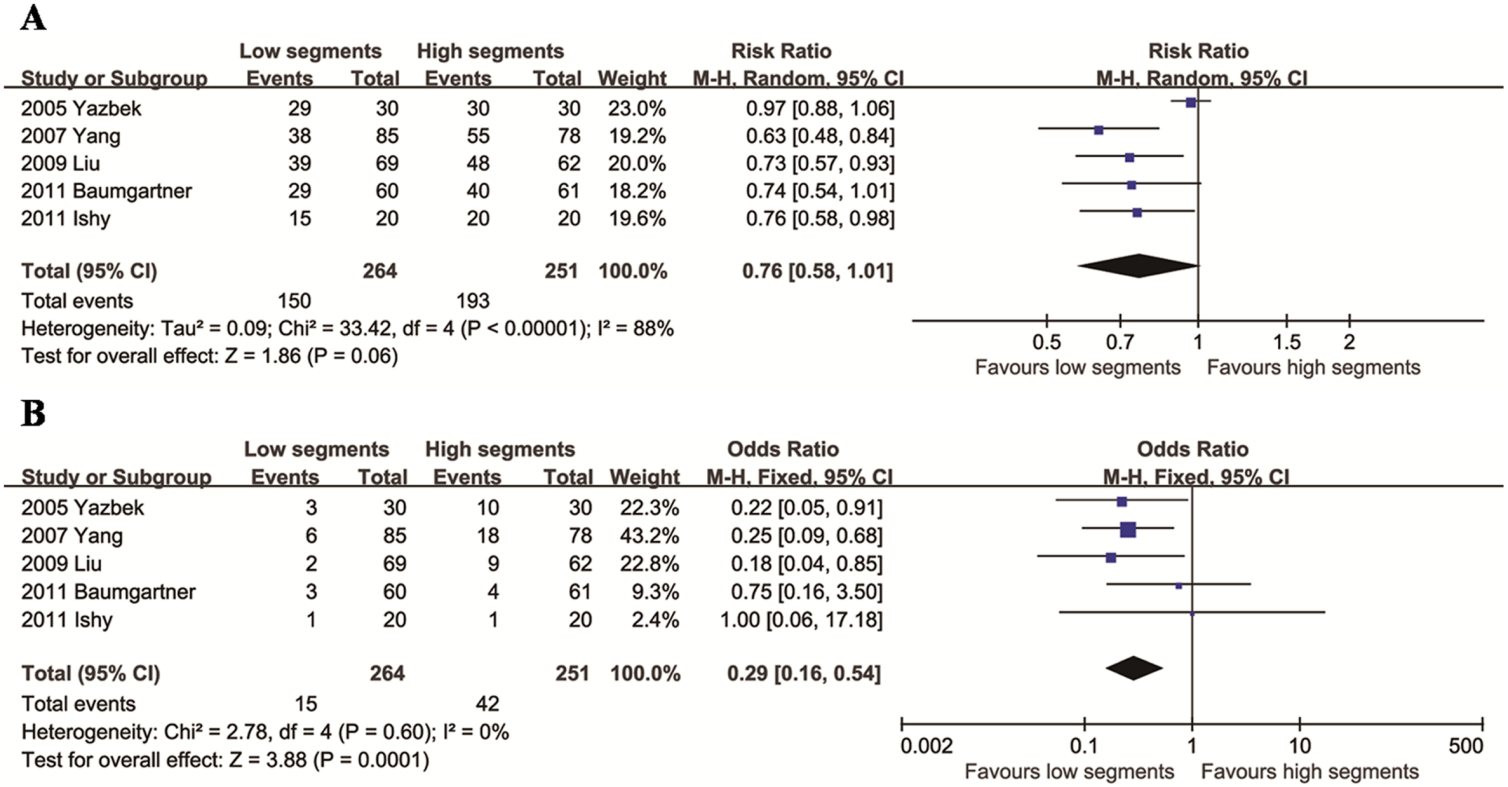
Relative risks for total CH (A) and moderate/severe CH (B) in the low segments group compared to the high segments group.

We identified five articles that compared VTS of low and high segments (if T2 vs T3, T2 is the high segement and T3 is the low segment; if T3 vs T4, T3 is the high segement and T4 is the low segment). The mean difference in total CH between the two groups was not significant (95% CI: 0.58 to 1.01, *p* = 0.06), with significant heterogeneity across studies (*p* < 0.0001; *I*^2^ = 88%, Fig 6A). There was no evidence of heterogeneity between these studies in the comparison of CH degree (*p* = 0.60, *I*^2^ = 0%). The results showed a lower degree of CH in the low segment group (*p* = 0.0001, Fig 6B).

### Publication Bias

**Fig 7:**
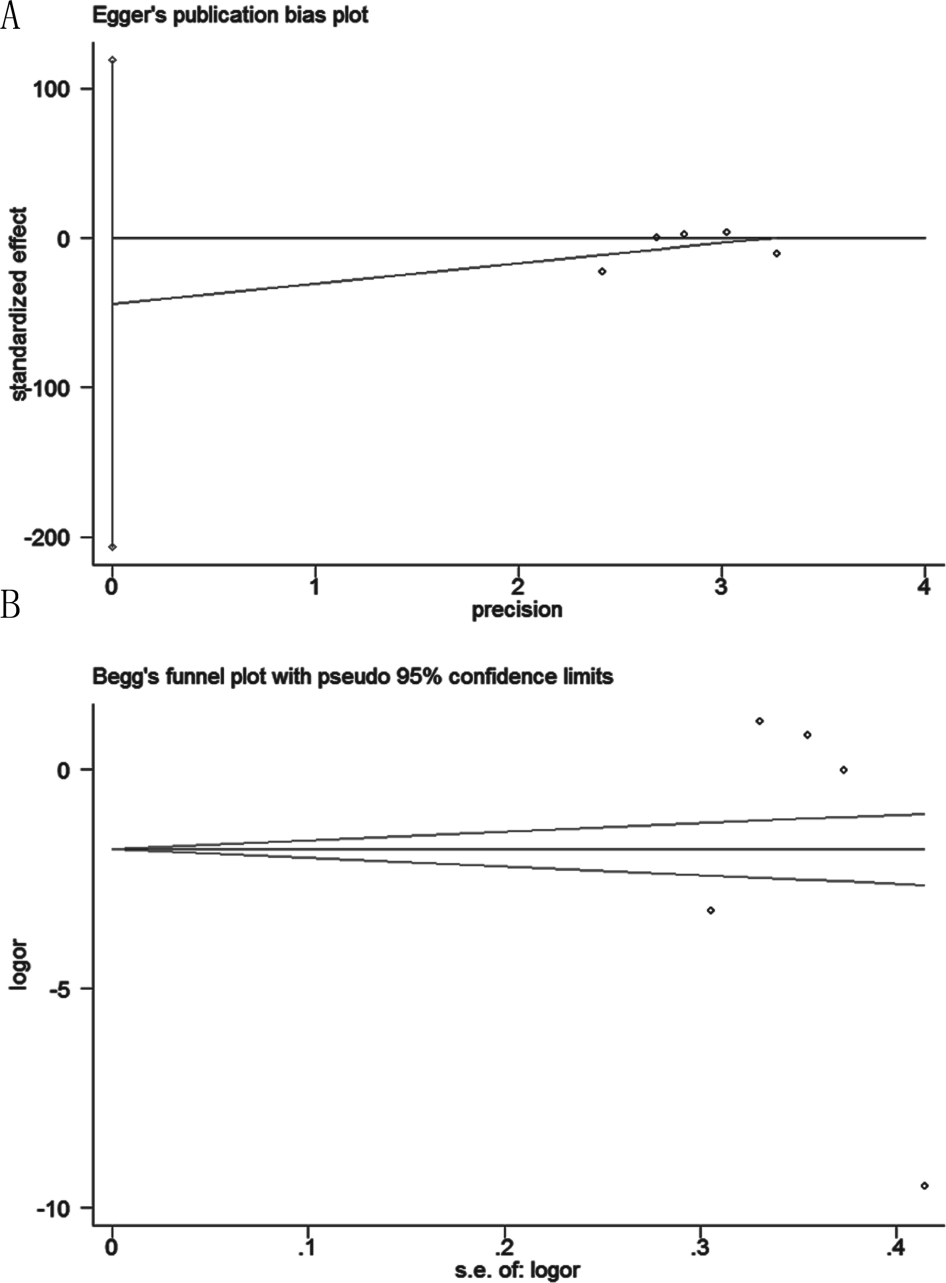
Begg's funnel plot and Egger's test risk for T3 segment, suggesting no publication bias existing in the pooled analysis. (A). Egger’s test, *p* = 0.452; (B). Begg's test, *p* = 0.462.

Assessment of publication bias using Egger’s and Begg’s tests showed that no potential publication bias existed among the included trials (Egger’s test: *p* = 0.452; Begg’s test: *p* = 0.462) (Fig 7).

## Discussion

VTS is now recognized as effective treatment of severe PH, and the development of an easy and safe video-assisted procedure has allowed for rapid spread of its application. VTS has been applied to various segments with different results. Traditionally, the T2 ganglion was regarded as the key pathway for the hands and was the most common site of VTS [16, 17]. However, a high incidence of severe CH affected quality of life after surgery [4, 8, 18]. Gray reported that the preganglionic fibers to the arm originate mostly from the third to the sixth spinal segments and the third and fourth segments were considered as the main lesions [19]. The results of a study that compared VTS of the T2 and T3 suggest that avoiding the T2 ganglion might decrease the risk of CH [8]. Both T3 and T4 VTS have been reported in the past several years [12, 20, 21, 22], resulting in fairly good results and a varying incidence of postoperative CH. At present, there remains a lack of expert consensus for the surgical procedure regarding the extent of denervation.

The present study included a total of 1200 patients with PH, providing the most comprehensive evidence for segment selection of VTS. Our results showed that more moderate/severe CH was found after VTS of multiple segments and high segments. This result is similar to that found in a study which suggests that VTS of these segments could increase the incidence and severity of postoperative CH [6].

In the single segment subgroup, our results showed no significant differences between T2/T3 VTS and other segments in terms of both total and degree of CH. T4 VTS was better at limiting CH compared to other segments. By chance, all studies in this review included only T4 and T3. It could therefore be considered as evidence that VTS of the T4 segment offers a better chance of avoiding postoperative CH than T3 segment. Lin *et al* confirmed by nervous tract mapping that neither T2 nor T3 are the major sympathetic innervation to hands and this role is performed by T4 and the lower ganglia; they also showed that only a small portion of the T3 sympathetic tone influences hand sweating. The authors therefore put forward the "Lin-Telaranta classification" that blocking the T4 thoracic ganglion was suitable for PH with or without axillary hyperhidrosis [23].

The possible limitations of our study must be considered when interpreting the findings. First, only eight RCTs were included in the study, which might have weakened the quality of the results. Second, there was no uniform standard for evaluating the severity of postoperative CH, which might increase the heterogeneity between studies. Third, the conclusion was proved by three indirect evidences, which might decrease the credibility of the conclusion. Fourth, the evaluation time of postoperative CH was not consistent in the reviewed studies, which might decrease the comparability of data.

## Conclusion

VTS of high segments or multiple segments could increase the incidence and severity of postoperative CH. T4 seems to be the best segment for VTS as the surgical treatment of PH. However, due to possible bias in the original studies, inter-study heterogeneity and the inherent limitations of our meta-analysis, this conclusion requires further validation by more high-quality and large-scale RCTs.

## Supporting Information

S1 File PRISMA checklist. (DOC)
